# FDG–PET findings associated with various medical procedures and treatments

**DOI:** 10.1007/s11604-022-01376-w

**Published:** 2022-12-28

**Authors:** Chio Okuyama, Tatsuya Higashi, Koichi Ishizu, Tsuneo Saga

**Affiliations:** 1 Division of PET Imaging, Shiga Medical Center Research Institute, 5-4-30, Moriyama-cho, Moriyama City, Shiga 524-8524 Japan; 2 Department of Molecular Imaging and Theranostics, National Institutes for Quantum Science and Technology, Chiba, Japan; 3 Human Health Sciences, Graduate School of Medicine, Kyoto University, Kyoto, Japan; 4 Department of Advanced Medical Imaging Research, Graduate School of Medicine, Kyoto University, Kyoto, Japan

**Keywords:** FDG–PET, Medical procedures, Adverse effect, Physiological reaction, Physiological effect

## Abstract

[^18^F]-fluorodeoxyglucose (FDG) positron emission tomography (PET) is a well-established modality with high sensitivity for the diagnosis and staging of oncologic patients. FDG is taken up by the glucose transporter of the cell membrane and becomes trapped within the cell. In addition to malignant neoplasms, active inflammatory lesions and some kinds of benign tumors also accumulate FDG. Moreover, the degree of uptake into normal organs and tissues depends on various physiological conditions, which is affected by various medical procedures, treatments, and drugs. To avoid misleading interpretations, it is important to recognize possible situations of unexpected abnormal accumulation that mimic tumor lesions. In this review, we present various FDG findings associated with surgical or medical procedures and treatments. Some findings reflect the expected physiological reaction to treatment, and some show inflammation due to prior procedures. Occasionally, FDG–PET visualizes other disorders that are unrelated to the malignancy, which may be associated with the adverse effects of certain drugs that the patient is taking. Careful review of medical records and detailed interviews of patients are thus necessary.

## Introduction

Fluorine-18 fluorodeoxyglucose (FDG) positron emission tomography (PET) or PET/computed tomography (PET/CT) is a clinically accepted modality with high sensitivity for detecting malignant neoplastic lesions. However, increased glucose metabolism with FDG accumulation is not only specific for neoplastic lesions but is also observed in many non-tumorous sites. Such sites include pathologically benign lesions, especially inflammatory lesions, and normal organs, because most cells require glucose for energy supply. Various medical procedures and prior treatments cause unexpectedly increased accumulation that is not associated with neoplasms.

These FDG findings may reflect specific drug effects that patient and clinician can expect. FDG–PET also sometimes presents unexpected reactive findings that can be associated with previous procedures. Certain adverse effects and drug-associated diseases can be visualized on FDG–PET. In this review article, these iatrogenic FDG findings were addressed using relevant medical procedures or treatments.

## Control of blood glucose

FDG, as well as glucose, is taken up by cells from circulation through cell membrane glucose transporters (GLUT) and is phosphorylated into FDG-6-phosphatate by hexokinase, the first enzyme in the glycolysis pathway. FDG-6-P is trapped within cells, because it is a poor substitute for glucose-phosphate isomerase, an enzyme involved in the second step of glycolysis. Considering glucose metabolism, muscles play a unique role in glucose storage, in the form of glycogen, and supply glucose for metabolic demands. GLUT-4, the main GLUT in the myocytes of the skeletal muscle and myocardium, usually stays within myocytes and is transferred onto the cell membrane when glucose transport is needed.

### Insulin

**Fig. 1:**
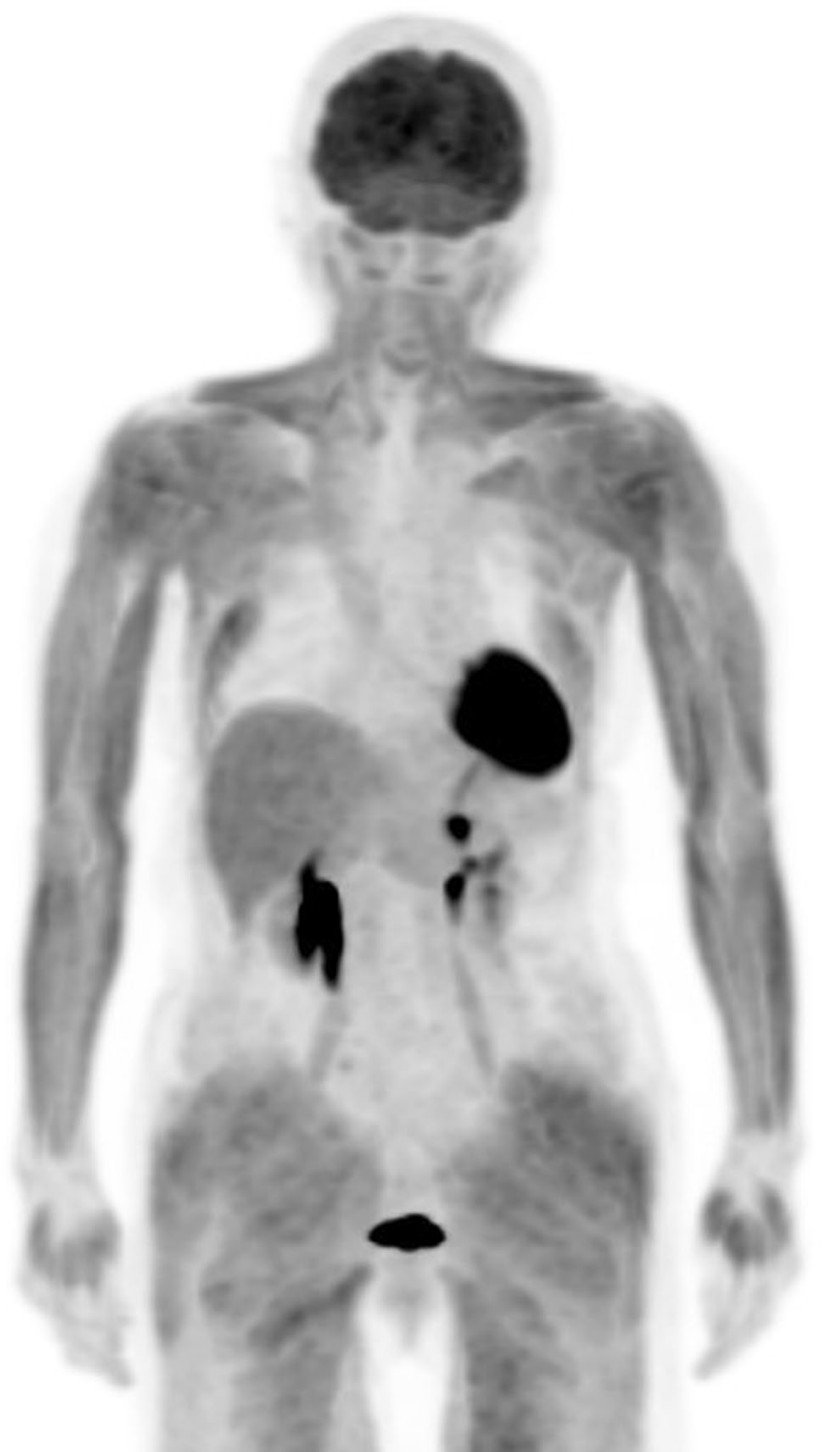
Diffuse increased accumulation in the muscles of a patient who had consumed a meal 2 h before FDG injection. An insulin injection before FDG administration causes a similar image

Insulin induces GLUT-4 transfer onto the cell membrane of myocytes to import glucose from the blood, after which glucose is stored in the myocytes as glycogen, which decreases excessive blood glucose levels. Therefore, more than 4 h of fasting and careful scheduling of insulin use before FDG administration are required [1]. In contrast, intrinsic insulin secretion and extrinsic high insulin levels induce strong FDG uptake in myocytes, which results in images that are unsuitable for evaluating tumor distribution with diffusely increased muscular uptake [2] (Fig. 1).

### Increased intestinal uptake caused by metformin

**Fig. 2:**
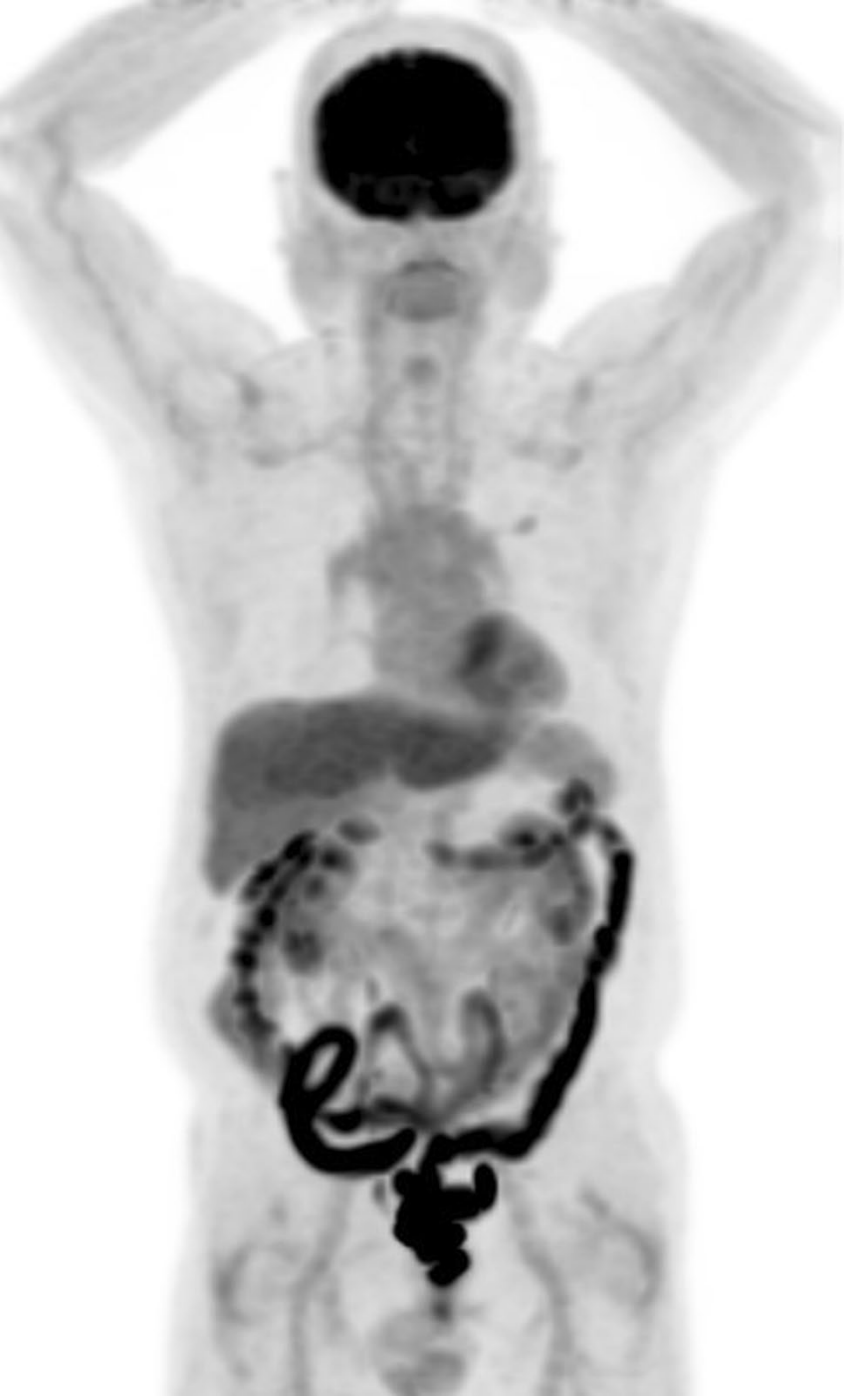
Increased bowel FDG distribution in a patient using metformin

Gontier et al. first found that increased intestinal FDG uptake is commonly observed in patients with type 2 diabetes mellitus who are using metformin [3] (Fig. 2). Increased intestinal FDG uptake can obscure intestinal lesions, and significant findings may be missed. Therefore, the effect of metformin discontinuation on intestinal FDG uptake has been studied [4, 5]. These studies showed that FDG uptake in both small and large intestines decreased a few days after metformin withdrawal. Recently, Morita et al. used PET/MRI, which enables simultaneous acquisition of metabolic information with PET and morphological information with MRI. They clarified that FDG levels in the lumen of the intestinal tract is significantly greater in patients treated with metformin than in those not treated with the drug [6, 7]. However, the amount of FDG in the intestinal wall did not significantly differ between the two groups. Their discovery using PET/MRI has provided new insights into the mechanism of action of this drug.

## Invasive or surgical procedures

**Fig. 3:**
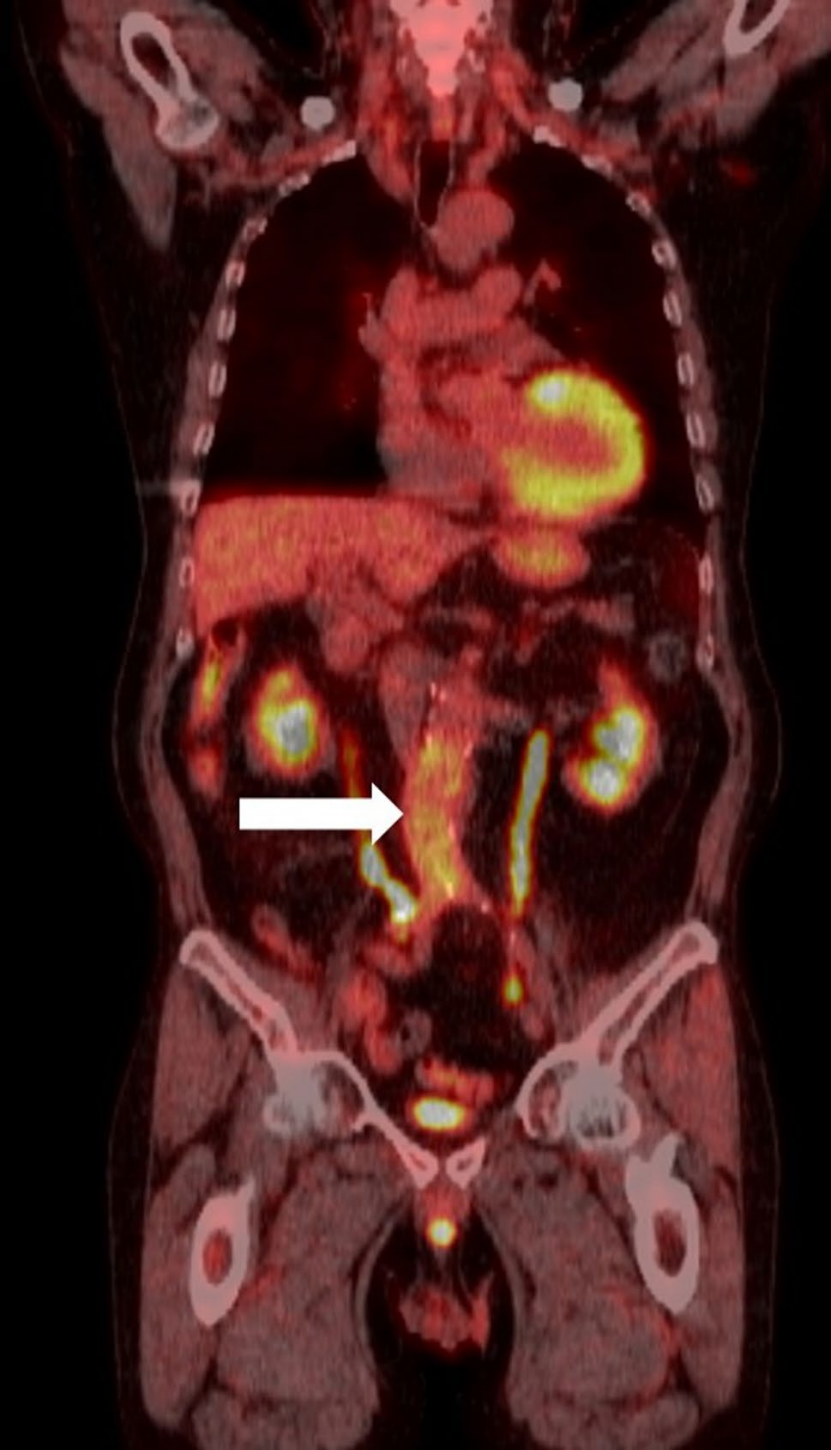
FDG accumulation along the abdominal aortic graft wall (arrow) is seen, without any particular findings indicating active inflammation, 3 years after operation

**Fig. 4:**
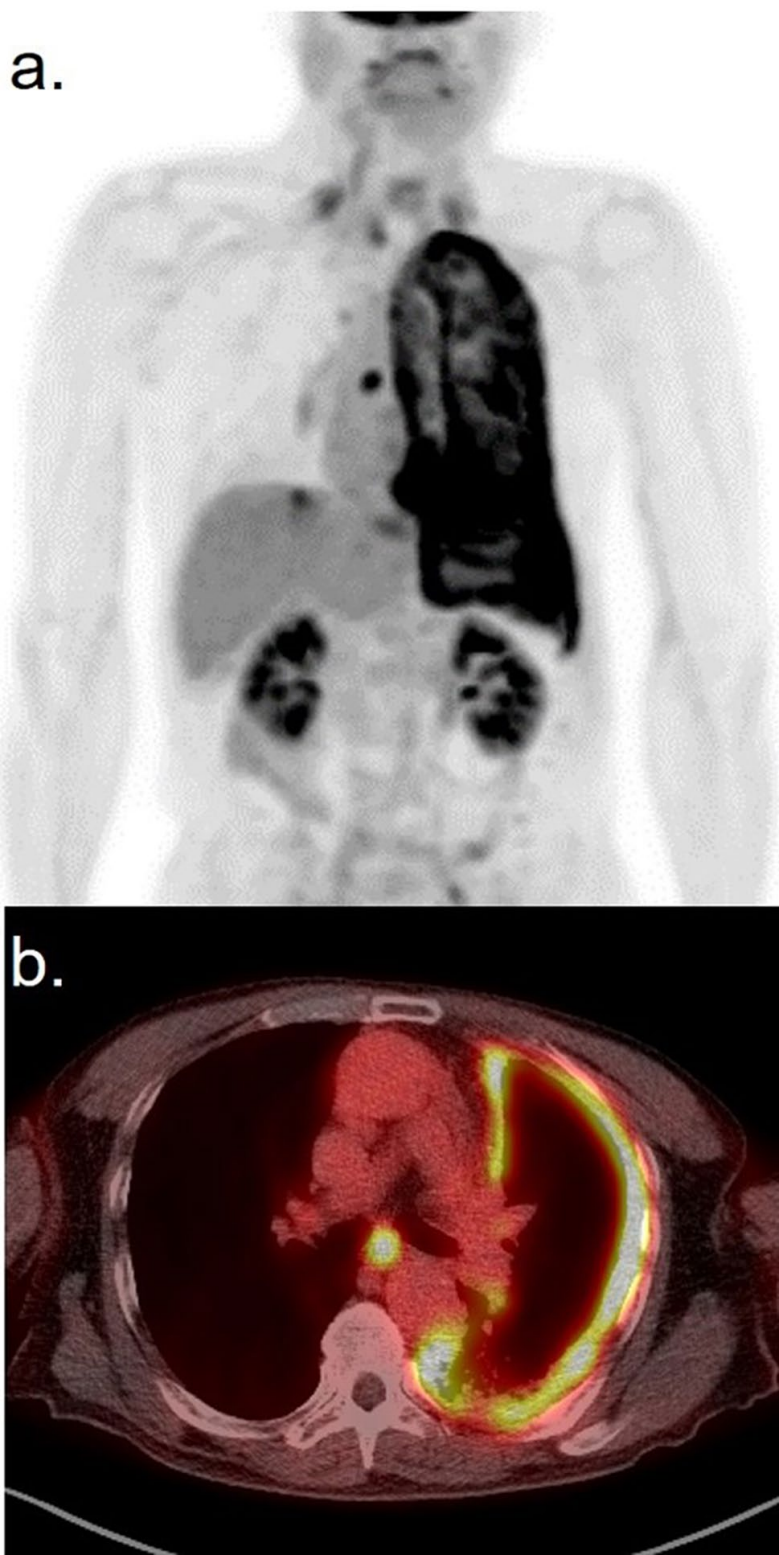
Intense FDG accumulation spreading inside the left pleura 1 month after pleurodesis with OK-432 after bruising the chest for pleuritis carcinomatosis. **a** MIP, **b** axial fusion image)

The abnormal FDG accumulation associated with inflammatory processes is often observed after invasive procedures, such as at surgical incision lines, along the drainage tube, around the stoma, or at the tracheostomy site. Although aortic graft infections are visualized as intense focal accumulation, mild-to-moderate uptake along the graft is normally seen for years, even without infection (Fig. 3). Pleurodesis with inflammation-inducing agents, such as OK-432 or talc, results in high FDG uptake [8, 9] (Fig. 4). Nishimori et al. reported that FDG uptake in non-malignant inflammation after pleurodesis with OK-432 appeared linear, while malignant lesions showed nodular and higher accumulation than benign lesions [10].

Since clinicians usually know most of these medical histories, it is not difficult to correctly interpret FDG uptake due to iatrogenic inflammation. However, there are some situations in which they do not recognize the history, such as sclerotherapy of the hemorrhoid, silicone injection for augmentation of the breast or buttock [11], or other cosmetic facial fillers [12, 13] performed at other institutions. Patients often think that these medical procedures are unrelated to their neoplasm and may not have provided this information. Interrogating the patient is as important as reviewing medical records to avoid misleading management due to incorrect interpretation of focal abnormal accumulation after these procedures.

## Repeated subcutaneous injections

Repeated subcutaneous injections often cause granuloma formation or reactive induration with increased FDG uptake at the injection sites [14]. This is often observed in patients with prostate cancer or breast cancer, who have been receiving luprorelin acetate (an LH–RH analog) therapy, or in patients with diabetes receiving insulin injections.

## External radiotherapy

Referring to the radiotherapy plan, images are important when interpreting FDG–PET results for patients who received radiotherapy.

External radiotherapy affects healthy organs within the irradiated area near the tumor. The physiological function of the bone marrow, brain, or tonsils within the irradiated area is impaired, and FDG accumulation in the corresponding area is usually low compared with the surrounding healthy area. Decreased vertebral uptake is frequently observed in the thoracic vertebrae in patients with esophageal cancer, lung cancer, or other mediastinal tumors and in the lumbar vertebrae and sacrum in patients with uterine or ovarian cancer. Although interpreting the post-irradiation finding for symmetrically decreased uptake is not difficult, it is sometimes difficult to interpret asymmetrical uptake in the bones or palatine tonsils after irradiation on one side. Because physiological accumulation in the bone or tonsils usually shows a wide range, careful consideration is required to judge whether the area with higher uptake is abnormal or whether the area with lower uptake indicates impaired function related to irradiation.

**Fig. 5:**
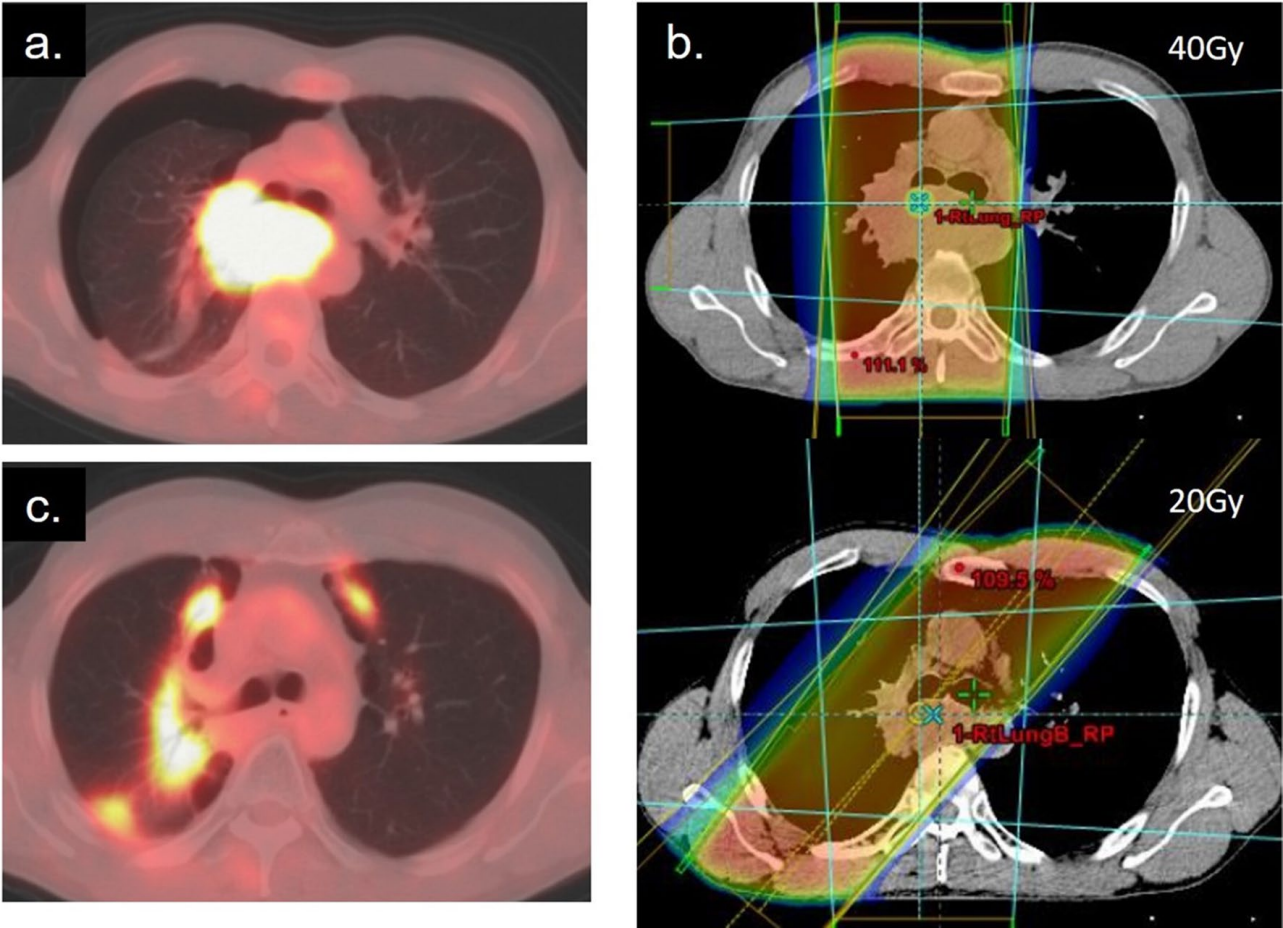
Axial images of a case of radiation pneumonitis that occurred in a patient with esophageal cancer. **a** FDG–PET/CT before treatment, **b** image of the irradiation field with two radiation direction (1. 40 Gy (upper) and 2. 20 Gy (lower)), **c** FDG–PET/CT 11 months after radiotherapy

**Fig. 6:**
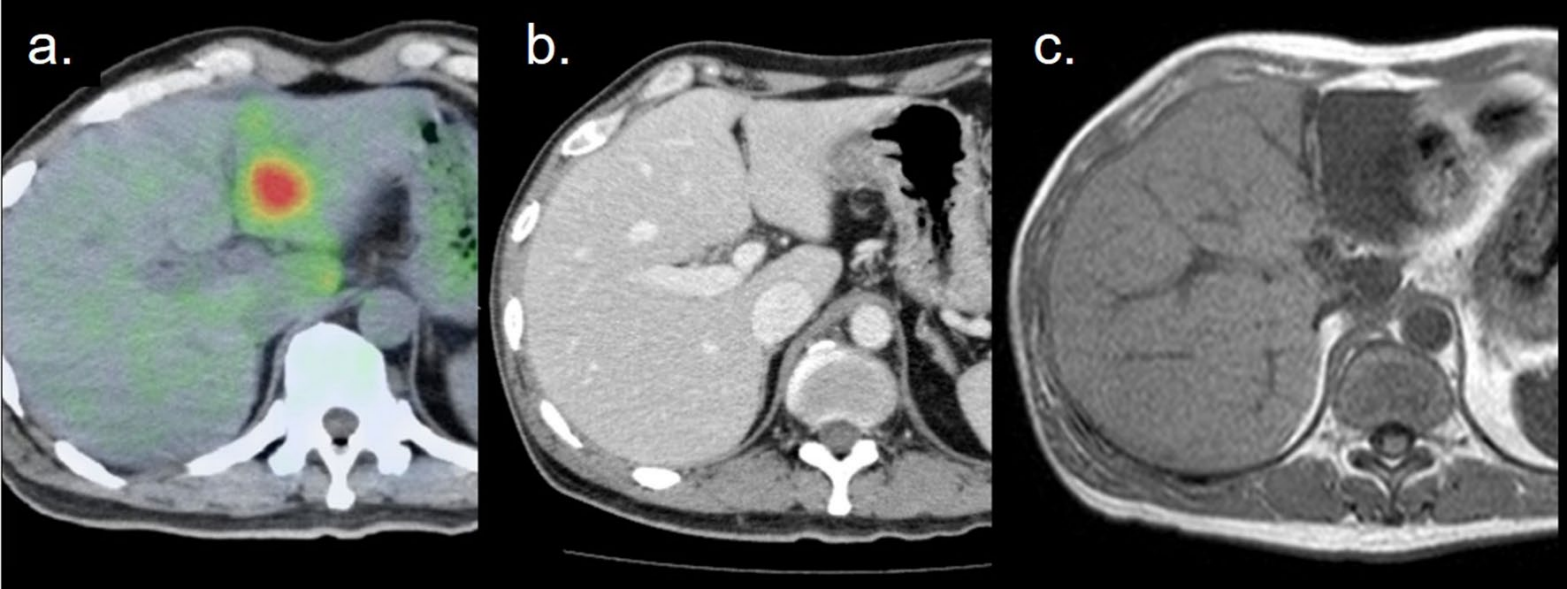
Images of a patient with testicular cancer who had undergone radiotherapy for a metastatic bone tumor at his L1 spine 2 months previously [17]. The FDG–PET/CT showed a localized hot spot in the lateral segment of the left lobe of the liver (**a**). Although the contrast-enhanced CT performed 10 days previously showed no abnormality (**b**), the MRI performed 7 days later showed a well-bordered square low signal area indicating radiation-induced hepatitis (**c**). (Partly cited from reference #17)

External radiotherapy also causes localized inflammation within the field. Radiation pneumonitis frequently occurs in cases of esophageal cancer, lung cancer, mediastinal tumor, or breast cancer and requires steroid therapy [15] (Fig. 5). Sharply margined increased FDG uptake is seen in the abnormal attenuation area, and the distribution is irrespective of the anatomical lobe or airway section. In the early stage, only FDG uptake was observed, without abnormalities in the CT images. The soft tissue, including the muscle or subcutaneous fat in the irradiated area, also has increased FDG accumulation to some extent. Radiation-induced hepatitis is visualized as a well-defined increased FDG accumulation corresponding to the radiation field, which is not always accompanied by abnormalities on plain CT images of PET/CT [16] [17] (Fig. 6).

Radiation-induced myocardial damage with focally increased FDG uptake has also been reported in patients with thoracic esophageal, lung, or breast cancer [18]. The physiological uptake in the myocardium varies depending on the patient and the duration of the fasting time before the FDG–PET examination. Jingu et al. showed that areas with high FDG uptake in the basal myocardium that corresponded to the irradiated fields indicated a damaged myocardium with low ^201^TlCl and ^123^I-BMIPP uptake, delayed Gd-enhancement, or hypokinesia on cine-MRI studies [18].

## Chemotherapy

Intensive chemotherapy causes time-dependent changes in the physiological distribution of FDG in some organs. Diffuse homogeneous hyperaccumulation in the red bone marrow is commonly observed as a normal hematopoietic response for a few months after marrow suppression due to intensive chemotherapy [19, 20].

The thymus, which has a high metabolic activity in the first years of life, gradually decreases in size with age. In pediatric, adolescent, and young adult patients, uptake in the thymus shows serial changes in association with physiological reactions after chemotherapy. The thymus, which shrinks during or soon after chemotherapy, shows hyperplasia as an immunological rebound phenomenon, which is characterized by lymph follicles with large nuclear centers and the infiltration of plasma cells following chemotherapy-induced inhibition of lymphocyte proliferation [21]. This rebound phenomenon occurs slightly later than that in the bone marrow. Symmetrical enlargement is accompanied by intense FDG uptake, which reaches a peak 10 months after the cessation of chemotherapy [22].

A similar dynamic change is also observed in the tonsils; the accumulation is low during intensive chemotherapy and then increases after the completion of chemotherapy. The high accumulation persists for a long period, as in the thymus. High tonsillar accumulation may be associated with reactive enlargement, but it is not always accompanied by a morphological change [23].

**Fig. 7:**
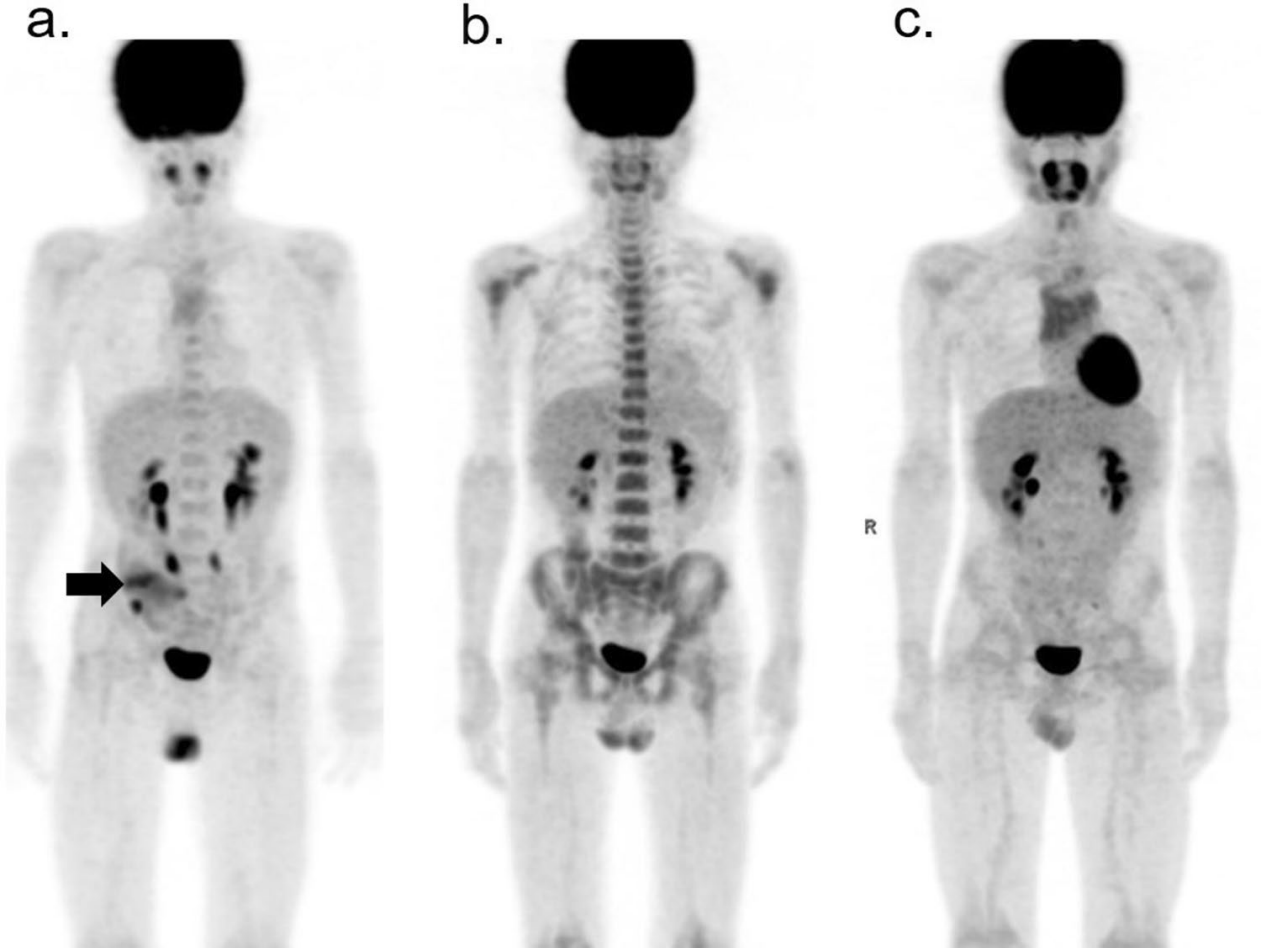
Serial changes in physiological FDG uptake in a pediatric patient with lymphoma: **a** before treatment (arrow shows the primary spot), **b** 1 month after the end of chemotherapy, and **c** 1 year after treatment. The image **b** shows diffusely increased uptake in the bone marrow. One year after chemotherapy, rebound uptake is seen in the thymus and tonsils, both of which seem to be enlarged. The bone marrow uptake has returned to its initial levels

Figure 7 illustrates the serial changes in physiological uptake in the bone marrow, thymus, and tonsils of a pediatric patient with lymphoma [23]. The areas where these serial reactions occur after intensive chemotherapy are also the sites, where lymphoma is frequently involved; therefore, the physician should take the time course of the patients’ treatment protocol into consideration so as not to jump to the conclusion that the high uptake area indicates recurrent tumors.

### Administration of G-CSF

**Fig. 8:**
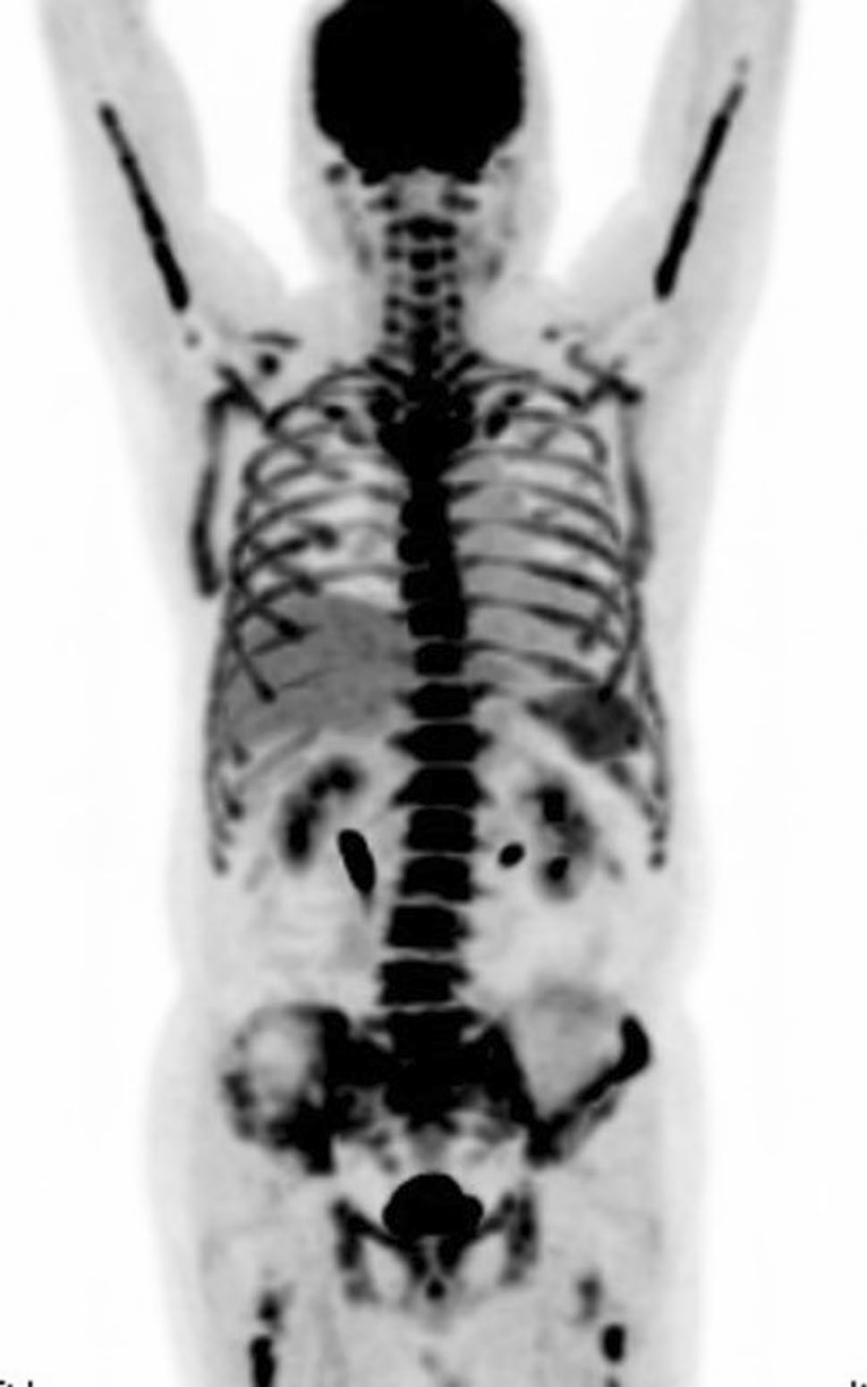
Marked increased uptake in the hematopoietic bone marrow and spleen in a patient with lymphoma, who received pegfilgrastim 7 days before the FDG–PET examination

Administration of granulocyte colony-stimulating factor (G-CSF) for leukocytopenia after chemotherapy increases physiological bone marrow FDG uptake, and Hanaoka et al. recommended an interval of 10 days after G-CSF administration to minimize its influence [24]. Recently, long-acting pegylated G-CSF (pegfilgrastim) has been frequently used, which causes consistent increased marrow uptake over a period of approximately 3 weeks following administration [25] (Fig. 8). An interval of at least 3 weeks after pegfilgrastim administration before PET/CT is now recommended.

## Vaccination

**Fig. 9:**
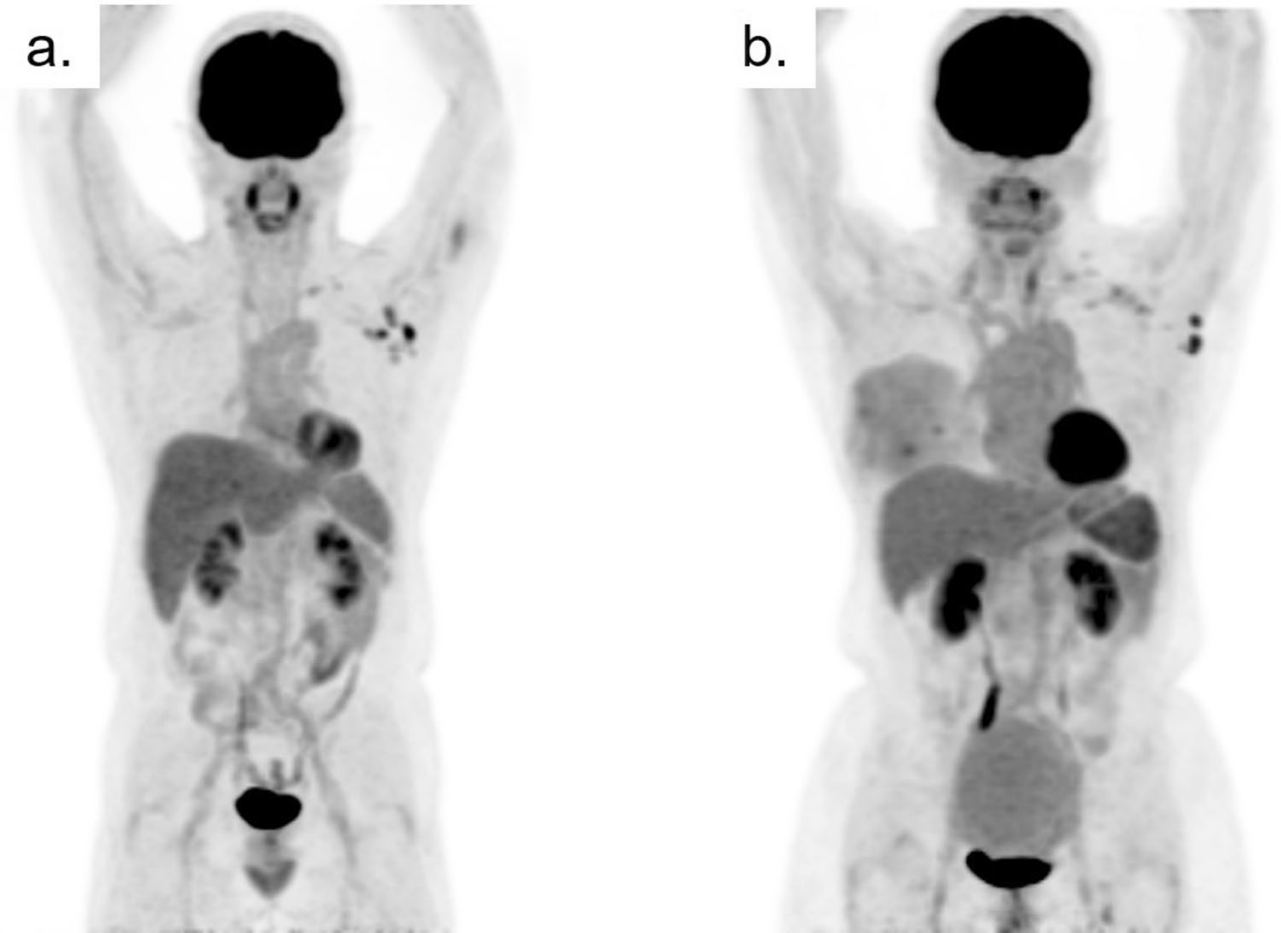
Two patients with high uptake in the left axillary and supraclavicular lymph nodes and spleen after vaccination: **a** 5 days after the second COVID-19 vaccination on the left deltoid muscle, **b** 6 days after subcutaneous influenza vaccination on the left arm in a woman with inflammatory breast cancer of the right breast

In these 2 years, transient FDG uptake in morphologically normal or slightly enlarged axillary, supraclavicular, and lower cervical lymph nodes after vaccination of the ipsilateral deltoid muscle have been commonly observed in the context of the COVID-19 mass vaccination [26–32]. It is sometimes accompanied by mild splenic enlargement with high uptake (Fig. 9a). These findings last for 4–6 weeks or longer after the most recent shot of the vaccine, and it has been recommended to ask patients for information about the date and sites of vaccination. It has also been recommended that oncologic patients, especially those with potential axillary or lower neck tumor involvement, such as breast cancer or head and neck cancer, be advised about the timing of the imaging and the site of vaccination [33].

Similar systemic immune responses to vaccination observed on FDG–PET have also been reported with various kinds of vaccinations, including seasonal influenza (Fig. 9b), pneumococcal, tetanus, diphtheria, pertussis, human papilloma virus, bacilli Calmette–Guerin (BCG), measles, smallpox, and anthrax [34–39].

Interestingly, regarding the third or following COVID-19 vaccination, the vaccine-associated hypermetabolic lymphadenopathy is mild and does not continue for weeks. Cohen et al. reported that uptake in the ipsilateral axillary lymph node persists only within the first about 5 days of administration and does not interfere with the interpretation of FDG–PET studies [40]. The mechanism of the immune response is considered to be related to the shortened duration of the reaction after the third vaccination. The first immune response of naïve cells is elicited by the first vaccine dose, and the amnestic response of memory B and T cells is induced by the second vaccine shot. Once memory cells have undergone clonal expansion, differentiation, and affinity maturation, the amnestic immune response requires only a short lag period after the third or later vaccination.

## BCG-induced granuloma

BCG is a vaccine for tuberculosis and sometimes causes transient vaccine-associated lymphadenopathy after shots. In addition, granuloma formation with high FDG uptake is sometimes observed after procedures using BCG. In pediatric patients, particularly those with impaired cellular immunity, multiple granulomas are formed in many organs, including the bone, liver, muscle, skin, and lungs, which may masquerade as disseminated neoplasms.

**Fig. 10:**
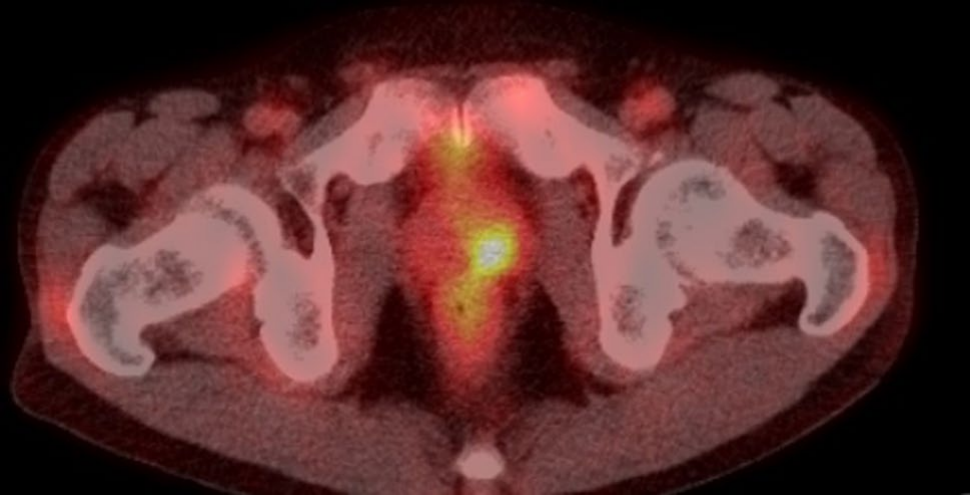
Local granuloma in the prostate in a patient who received intravesical BCG administration for bladder cancer

**Fig. 11:**
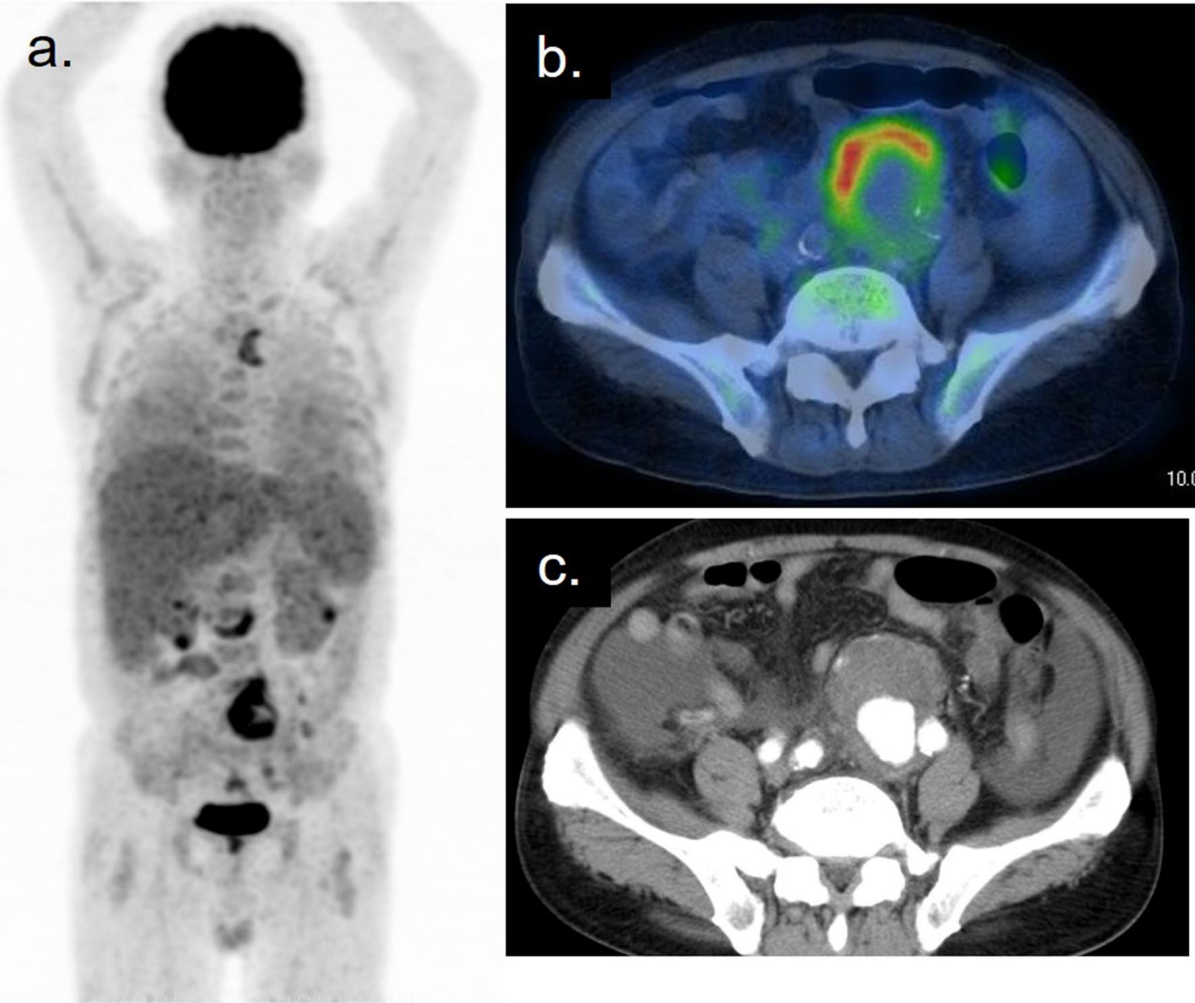
Ectopic granuloma at the abdominal aortic wall and disseminated granuloma in the lungs, liver, bone marrow, kidneys are visualized on the FDG–PET (**a**) [23]. The patient has a history of transurethral resection of bladder tumor and 8 times intravesical BCG injection for his bladder cancer. The axial PET/CT fusion image (**b**) shows intense accumulation on wall of the dilated left common iliac artery. Five days after the PET/CT examination, this patient developed an acute abdomen with impending rupture of this aneurysm (**c**)

BCG is also used as an immunotherapeutic agent that encourages the immune system to attack cancer cells. It is administered directly into the bladder of patients with non-muscular invasive bladder cancer. BCG-contaminated urine induces asymptomatic local granulomas anywhere along the genitourinary tract, kidneys, prostate, scrotum, and penis, which are visualized on FDG–PET in patients who received intravesical BCG therapy [41] (Fig. 10). Mycotic aneurysms sometimes occur as a progression of ectopic granulomas at the aortic or arterial walls [42, 43] (Fig. 11), which usually grow in a short period and often rupture. Occasionally, disseminated granulomas in the lungs, kidneys, bone marrow, liver, or skin have also been discovered in PET studies that mimic the systemic involvement of hematological disorders, such as lymphoma [44] (Fig. 11).

## Oii-LPDs

Patients with congenital or acquired immunodeficiency have a high incidence of lymphoproliferative diseases (LPDs). The WHO classification 2016 classifies immune-deficiency-associated LPDs into four subtypes: LPD associated with primary immunodeficiency disorders, lymphomas associated with HIV, post-transplant LPD, and other iatrogenic immunodeficiency-associated LPDs (Oii-LPDs) [45]. Oii-LPDs develop in patients treated with immunosuppressive drugs. The most common primary disease is rheumatoid arthritis, followed by dermatomyositis, psoriasis, psoriatic arthritis, systemic lupus erythematosus, and inflammatory bowel disease. Most cases involve patients using methotrexate (MTX); thus, the condition was first called MTX–LPD. However, patients treated with anti-tumor necrosis factor (anti-TNF) or other drugs have also reported, and it is now recognized that various drugs other than MTX can cause this disorder.

The pathological subtype of Oii-LPDs mainly consists of reactive lymphoid hyperplasia, polymorphic LPDs comprising infiltration of plasma cells, immunoblasts, and lymphocytes, and lymphomas, such as diffuse large B-cell lymphoma, Hodgkin lymphoma, Epstein–Barr virus (EBV)-positive mucocutaneous ulcer, or hepatosplenic T-cell lymphoma [46].

**Fig. 12:**
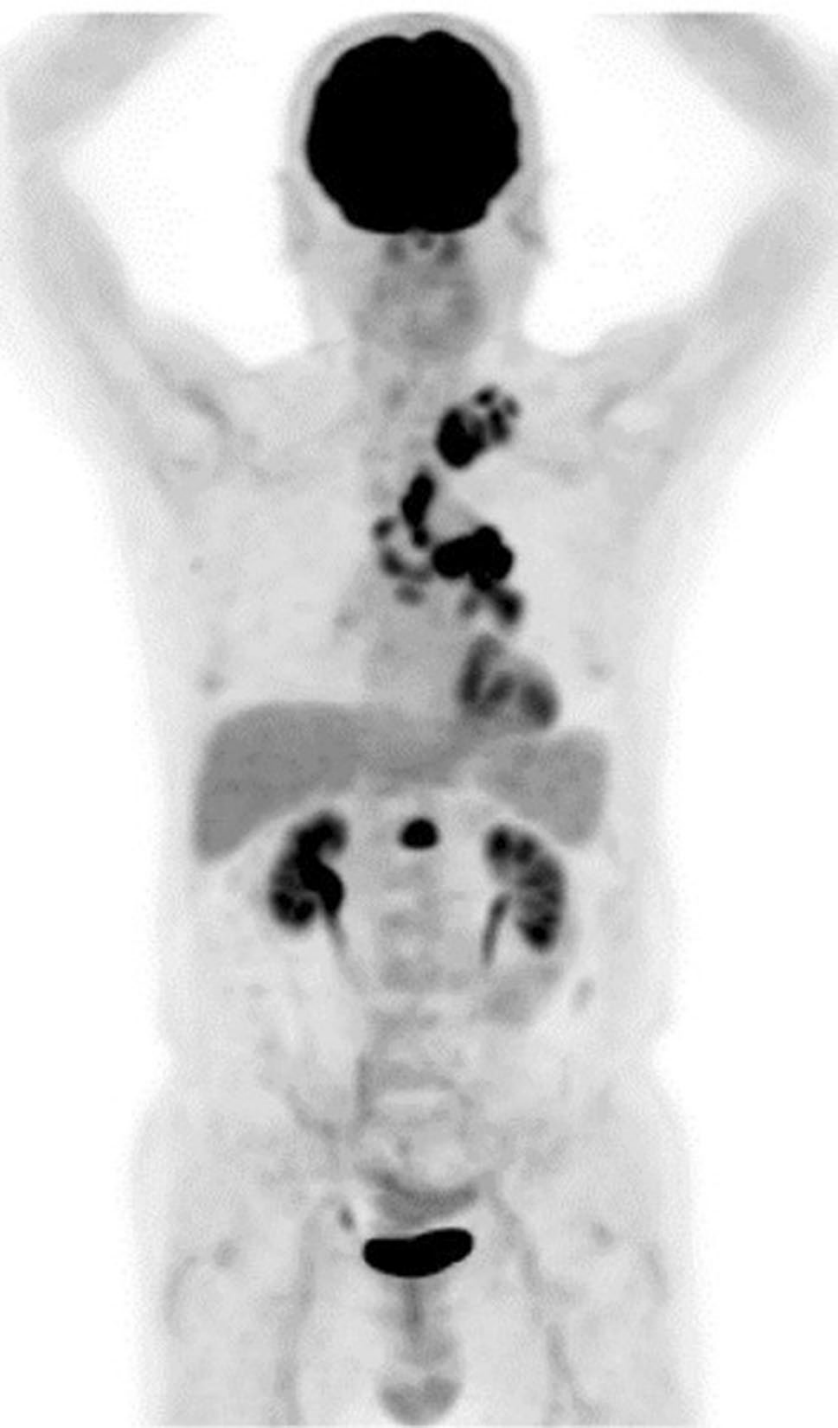
Patient with multiple lymphadenopathies that were diagnosed as Oii-LPD with positive EBV. He had been treated with MTX for rheumatoid arthritis. In this patient, most of the lymphadenopathies disappeared after MTX discontinuation

Although LPDs show similar FDG-positive lesions as lymphoma (Fig. 12), Oii-LPDs have some peculiar features, such as a high incidence of extranodal disease [47] and spontaneous regression. Approximately 70% of Oii-LPDs show spontaneous regression after the discontinuation of immunosuppressive drugs. Approximately 33% of patients who have experienced transient regression experience later relapse or recurrence [47].

EBV is a common gamma herpesvirus, and most of the world’s population is asymptomatically infected. Although EBV infects most healthy individuals without any pathogenicity, it primarily targets B lymphocytes and can develop lymphoid malignancies [48, 49]. EBV positivity is a major risk factor for Oii-LPDs, but EBV-encoded RNA positivity and higher lymphocyte counts in peripheral blood are predictive factors for its regression [47].

### EBVMCU

**Fig. 13:**
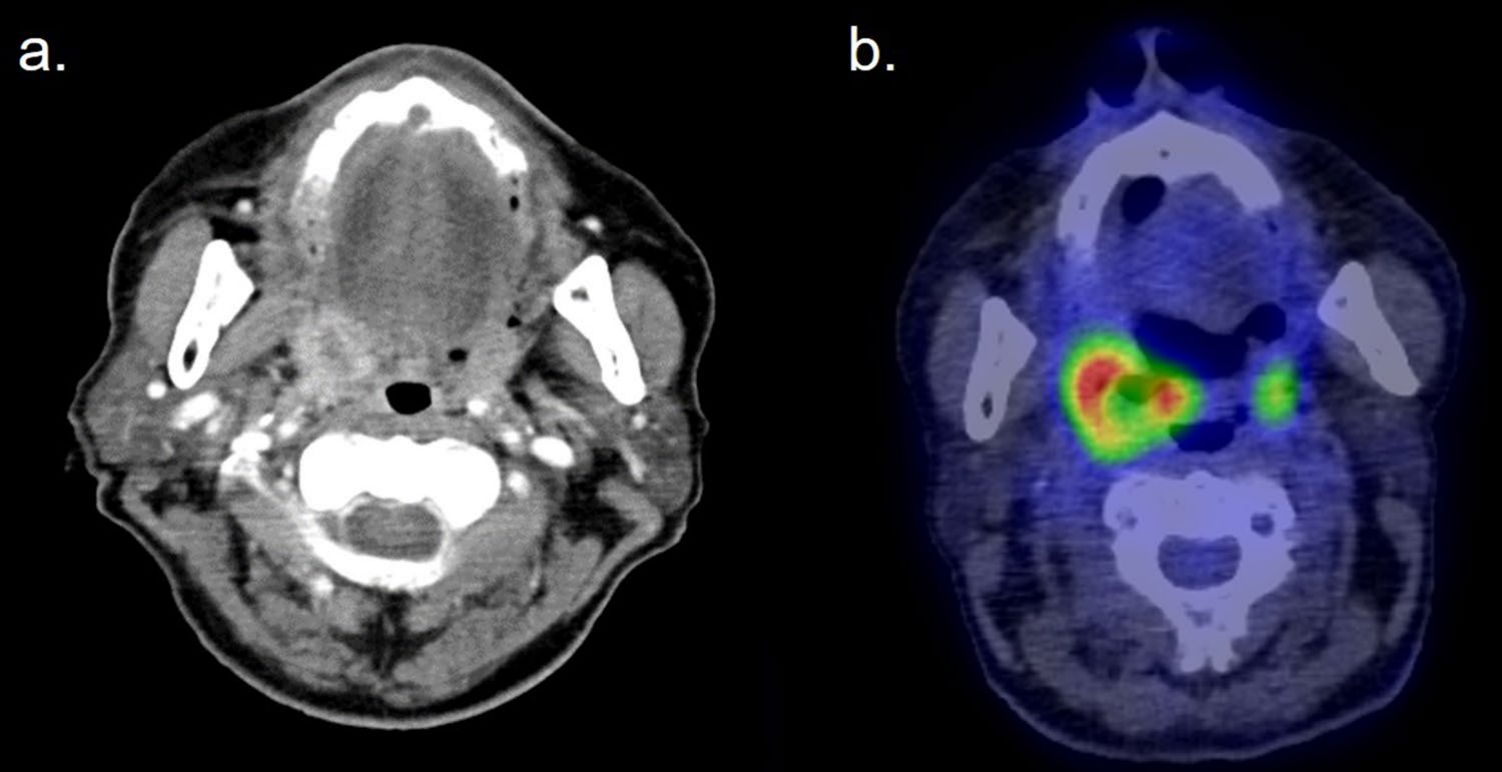
Patient with EBVMCU who had been receiving MTX for rheumatoid arthritis for 2 years. **a** Contrast-enhanced CT, **b** FDG–PET/CT fusion image

EBV-positive mucocutaneous ulcer (EBVMCU) is a recently recognized B-cell LPD that is driven by latent EBV infection and causes ulcerations in the oropharynx, gastrointestinal tracts, and skin [50, 51]. Intense FDG accumulation in the lesion mimics malignant tumors [52] (Fig. 13). This is considered to be a specific type of Oii-LPD that shows a relatively favorable prognosis. The lesions often disappear after the discontinuation of immunosuppressive drugs.

## Immunotherapy-related findings

**Fig. 14:**
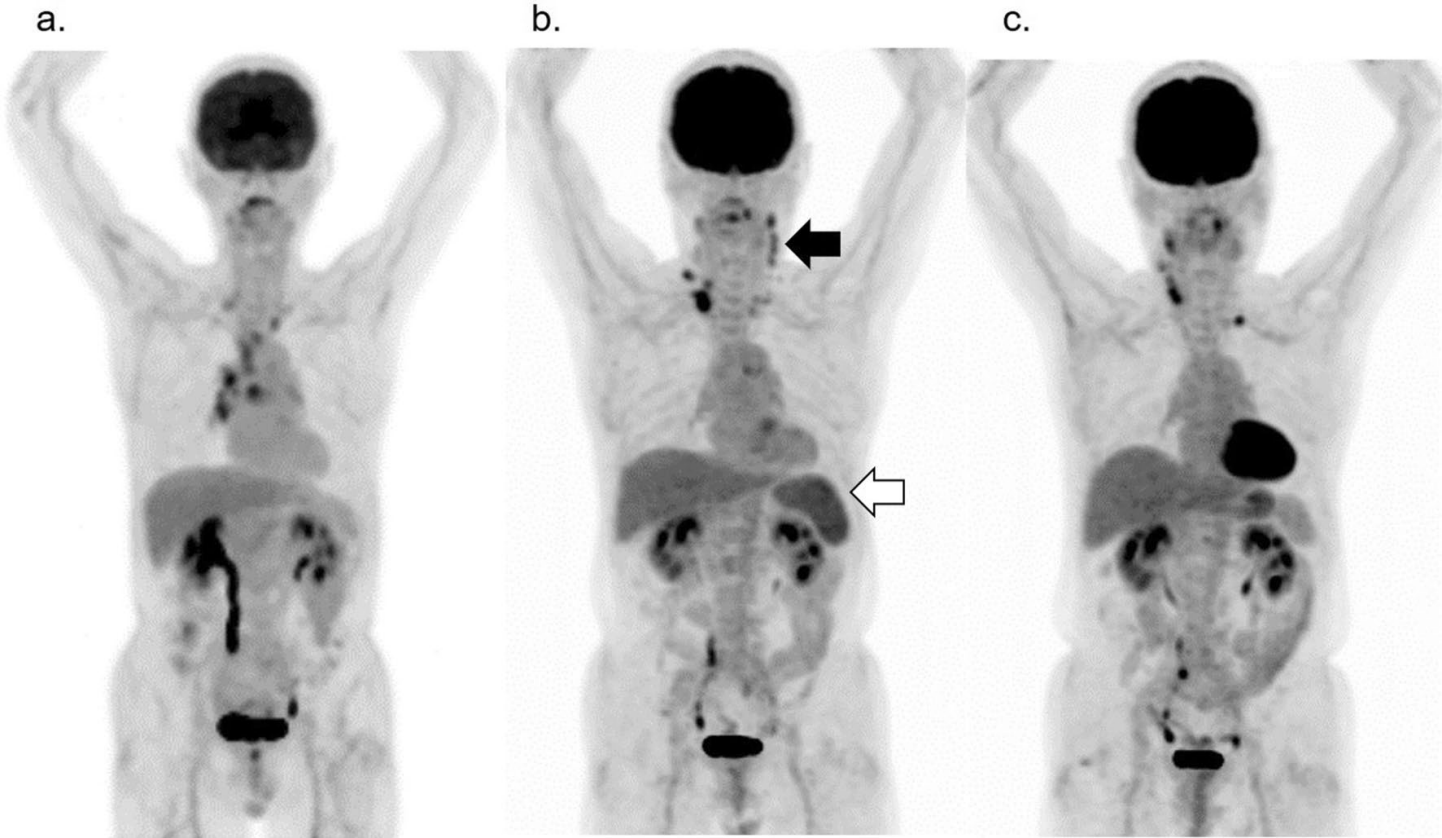
Patient with lung cancer with lymph nodes metastases (**a**). Six months after achievement of a complete response, lymph nodes metastases developed in the right lower neck and bilateral supraclavicular regions. The second PET scan (**b**), which was performed 3 weeks after starting anti-PD-L1, visualized another lymph node in the left neck (closed arrow) and increased uptake in the enlarged spleen (open arrow). The increased splenic uptake and lymphadenopathy in the left neck that had disappeared on the third PET scan (**c**) are considered immunotherapy-related findings

Immunotherapy has recently emerged as an important advancement in cancer treatment, in addition to surgery, radiation, chemotherapy, and molecular-targeted therapies. It is based on evidence that cancer development is enabled by the dysregulation of the immune system against neoplasms. Immunotherapy relies on the reactivation of the host immune system to recognize and kill cancer cells [53]; thus, FDG–PET often visualizes systemic immune activation after immunotherapy. FDG uptake in the bone marrow is attributed to inflammatory activity, and increased uptake in the lymphoid tissue, namely, in the multiple lymph nodes and spleen, is recognized as a marker of immunotherapy effectiveness [32, 54, 55] (Fig. 14).

Immunotherapy-related adverse effects (irAEs) are new toxicity profiles that involve many organs. Table 1 summarizes the FDG–PET findings of both immune reactions and irAEs. The pattern of adverse effects differs across immune checkpoint inhibitor classes and can be driven by different immune cell activation patterns. The early detection of irAEs and rapid intervention by systemic immunosuppression are important for improving patient outcomes. These events are not always associated with clinical symptoms and may only be diagnosed using imaging modalities. FDG–PET is frequently beneficial for the early identification of these events [56–58].

**Table 1:** Immunotherapy-related FDG positive findings

Disorder (possible symptoms)	CT findings associated with the increased FDG uptake
Reactive lymphadenopathy	Enlarged lymph node
Sarcoid-like reaction	Symmetrical mediastinal and hilar enlarged nodes
Reactive splenomegaly	Splenomegaly
Thyroiditis (Hyper/hypo-thyroidism)	Diffuse goiter/normal size
Pituitary hypophysitis (various symptoms due to hormone deficiency)	Enlarged pituitary gland
Colitis (diarrhea)	Intestinal mural thickening in a long segment with/without surrounding fat stranding
Pancreatitis	Swelling with/without peripancreatic fat stranding
Pneumonitis (cough, fever)	Non-specific findings of pneumonitis
Arthritis (arthralgia)	Non-specific finding of polyarthritis
Myositis (myalgia)	Non-specific

There have been many reports of patients who experienced apparent lesion progression but subsequent late responses to treatments, which has been termed “pseudoprogression”. Pseudoprogression has been frequently reported in patients with melanoma receiving anti-CTLA-4 treatment, whereas it is rare in other tumor types and with anti-PD1/PD-L1 treatment. Pseudoprogression is also observed in patients treated with CD19 specific chimeric antigen receptor T cell (CAR-T) therapy, which is also a new promising cancer immunotherapy that is now approved for the treatment of relapsed/refractory diffuse B cell lymphoma [59]. However, immunotherapy sometimes accelerates tumor growth, which is called “hyperprogression” [60]. Clinicians should interrupt the treatment if hyperprogression is suspected. Understanding these features and careful follow-up are required to assess the responses of patients to immunotherapy.

## MRONJ

**Fig. 15:**
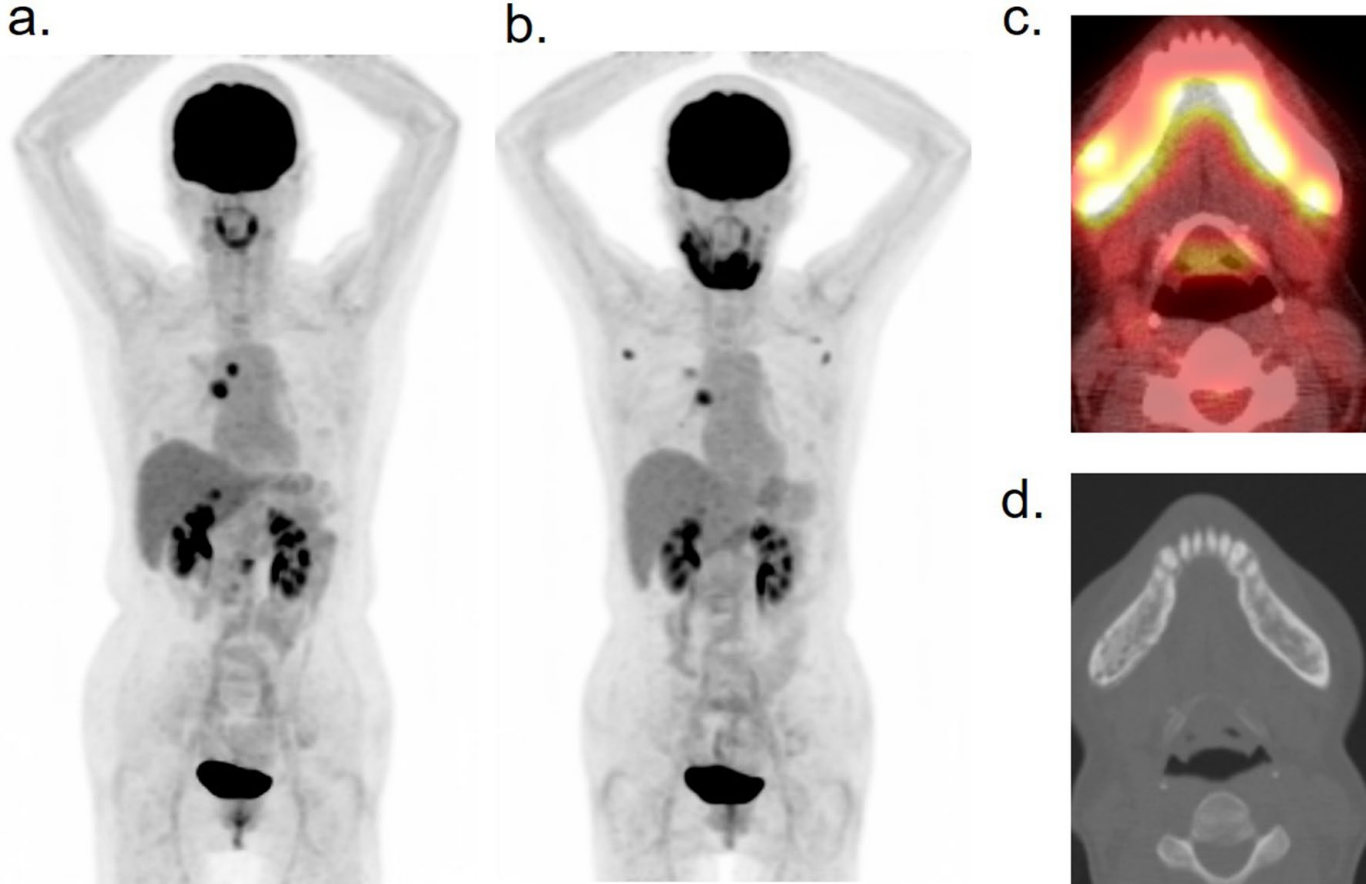
Patient with lung cancer with bone metastasis who developed medication-related osteonecrosis of the jaw due to bisphosphonate. The first PET scan (**a**) revealed metastatic lesions in the mediastinum, right adrenal gland, and a lumbar vertebra. The second PET scan detected the abnormality as an intense accumulation in the mandibular bone (**b**, **c**) earlier than the CT (**d**)

Chronic osteomyelitis or osteonecrosis of the jaw is one of the most intractable inflammatory conditions of the maxillofacial region. The incidence of medication-related osteonecrosis of the jaw (MRONJ) has recently increased. According to the American Association of Oral and Maxillofacial Surgeons, the definition of MRONJ includes all of the following criteria: (1) current or previous treatment with antiresorptive (bisphosphonate and denosumab) or antiangiogenic agents (bevacizumab and sunitinib); (2) exposed bone or bone that can be probed through an intraoral or extraoral fistula (e) in the maxillofacial region that has persisted for more than 8 weeks; and (3) no history of radiation therapy to the jaws or obvious metastatic disease of the jaws [61]. Jawbones are anatomically prone to osteomyelitis compared to other bones, because they face the oral cavity and are easily damaged by chewing food. FDG sometimes incidentally visualizes the inflammatory condition of the MRONJ [62, 63]. MRONJ should be included in the differential diagnosis when abnormally high FDG uptake is incidentally detected in the jaws of patients treated with the above-mentioned drugs (Fig. 15), even if the patients are asymptomatic in the early stages.

## Exercise

**Fig. 16:**
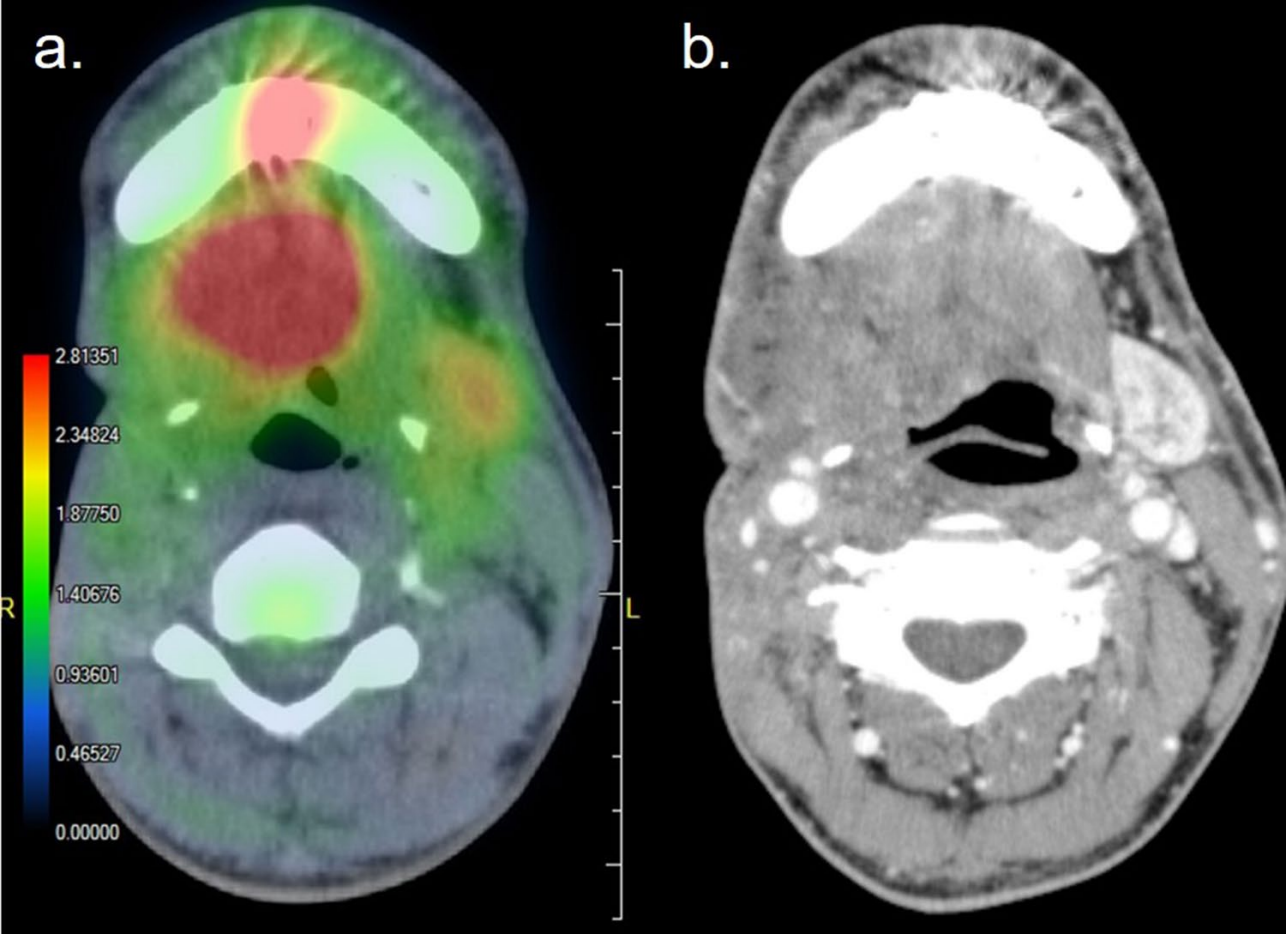
Patient with post-operative-state tongue cancer. An intense FDG accumulation seen in the center of the reconstructed tongue (**a**) without any abnormality on the contrast enhanced CT (**b**) or clinical inspection. The interview revealed that he had underwent rehabilitation for postoperatively impaired oral muscular function before the PET examination

Contracting skeletal muscles accelerates carbohydrate and fat metabolism during exercise. In muscles, where the redundant glucose is transformed into glycogen to be stored after meals, glycogen is quickly catabolized to glucose-6-phosphate for energy demand during exercise. However, glycogen storage in the muscle is insufficient for persistent exercise, and glucose uptake through GLUT-4 is activated. Exercise induces the transfer of GLUT-4 onto the cell membrane of myocytes to facilitate glucose uptake. By such a mechanism, FDG distribution in the body differs at rest and after exercise [64, 65]. After exercise, FDG accumulates in exercised muscles. For example, FDG uptake increases in the masticator muscles after chewing gum [66] and in the laryngeal muscles after speaking too much [67]. Therefore, for the evaluation of tumor metabolism, patients are asked to refrain from strenuous exercise and keep quiet, without speaking much, before FDG–PET examination. Postoperative patients with oral cancer undergo oral rehabilitation for impaired mastication, swallowing, or speaking. Patients may be unaware that they are exercising, because only localized muscles of a limited area around the mouth and larynx are trained. Hard exercise of the reconstructed tongue causes intense FDG accumulation after rehabilitation (Fig. 16), which may be misdiagnosed as a local recurrence.

## Miscellaneous drug-induced findings

### Activated BAT visualization due to β3 adrenergic agonist

It has been considered that physiological uptake in brown adipose tissue (BAT) is typically observed in pediatric or young adult patients, especially in the cold circumstances. Recently, apparent FDG accumulation in activated BAT by β3 adrenergic agonist, which is prescribed for overactive bladder in elderly patients, has been occasionally observed, even in the summer [68, 69]. As adrenergic β3 receptors exist on BAT as well as in the bladder detrusor muscles, β3 agonists sometimes cause intense FDG uptake in BATs. The characteristic distribution of the BATs that FDG visualizes in elderly patients activated by β3 adrenergic agonist or pheochromocytoma is seen in paravertebral or retroperitoneal spaces, while the BATs in pediatric or young adult patients are usually seen in the supraclavicular or lower neck regions. It is important to check the medication that patients are taking when BATs are visualized in unusual situations.

### Myocardial uptake influenced by medication other than insulin

GLUT-4 translocation to the muscular cell surface is, as described in the previous section, upregulated by high insulin levels. Moreover, in the myocardium, where the energy requirements are supplied by mainly fatty acids as well as glucose, and many factors including sex, age, obesity, and diabetes affect its glucose and fatty acid metabolism and influence on the myocardial FDG uptake [70]. The thyroid hormone levothyroxine upregulates GLUT-4 in the skeletal muscle but reduces the myocardial glucose uptake, and bezafibrate, which reduces serum triglyceride levels [71], cause increased myocardial uptake [72, 73]. Although the mechanism remains unclear, it is also reported the myocardial FDG uptake can be influenced by benzodiazepine.

### Drug-induced gynecomastia

Gynecomastia refers to the enlargement of male glandular breast tissue as a result of a hormone imbalance between estrogen and androgen. Although men aged > 50 years often experience physiological gynecomastia due to the decrease in testosterone levels with increasing age, gynecomastia may be indicative of an underlying health condition, including chronic renal failure, liver cirrhosis, thyrotoxicosis, malabsorptive states, and some particular tumors. In addition, drug-induced gynecomastia accounts for 10–25% of all gynecomastias, including estrogen, anti-androgen, antihypertensive, antifungal, antiparasitic, opioids, antiarrhythmic, proton-pump inhibitors, H2 blockers, dopamine receptor blockades, and anti-epileptic [74]. FDG–PET/CT shows soft tissue growth in the breast with increased tracer accumulation [75]. When enlargement occurs rapidly or is seen in one breast, a detailed history should be taken to determine whether the enlargement may result from a tumorous lesion.

### Others

FDG–PET can sometimes present various kinds of non-tumorous disorders that are non-specific but associated with some medications, of which patients may experience some involving symptoms or present abnormal laboratory findings.

Increased accumulation in the organs is seen in patients with drug-induced pneumonitis, nephritis, pancreatitis, or reactive lymphadenopathy, for which the physician can usually reach the diagnosis easily from clinical data. FDG–PET may visualize some rare drug-induced findings, For example, pegfilgrastim, a long-acting pegylated G-CSF, can induce vasculitis that causes antibiotic-resistant fever and high C-reactive protein levels in patients treated with pegfilgrastim for leukocytopenia due to chemotherapy [76]. Pegfilgrastim is also a causative agent of iatrogenic carotidynia, which is characterized by radiating pain and tenderness over the unilateral common carotid bifurcation. Since perivascular carotid inflammation occurs, FDG–PET reveals vasculitis in a limited area [77, 78]. Many kinds of drugs, including hypolipidemic agents (HMG-CoA reductase), antibiotics, major and minor tranquilizers, and anticancer agents, are known to cause drug-induced rhabdomyolysis. When FDG shows marked diffuse accumulation in the skeletal muscles, such as polymyositis [79] and the patient experiences muscle weakness and pain with increased serum creatine phosphokinase or myoglobin levels, the drugs that may cause rhabdomyolysis should be identified and stopped immediately to prevent serious renal failure.

Although these disorders are not specific, a thoughtful interpretation of FDG–PET could contribute to elucidating the cause of patients’ problems.

## Uncommon findings associated with FDG injections

### Stress during FDG injection

**Fig. 17:**
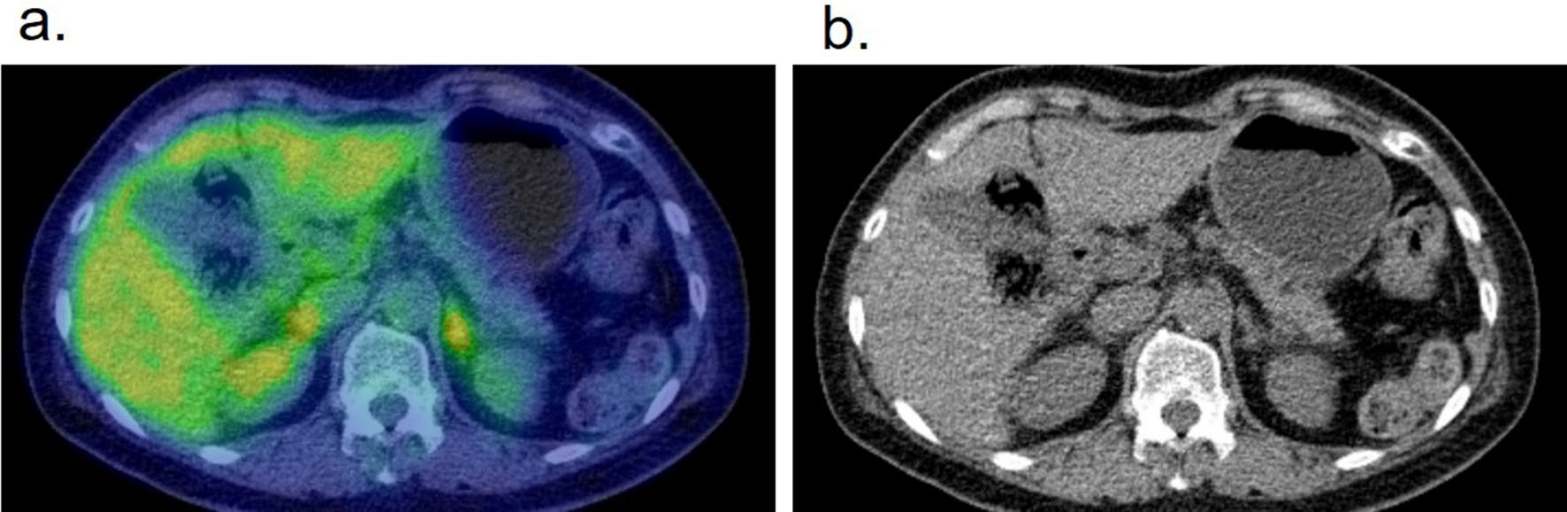
Increased FDG uptake in both adrenal glands with normal configuration in a case who developed vasovagal reflex at the FDG injection. **a** Fusion image, **b** CT

Adrenal medullary catecholamine production is considered the first response to stress and anxiety, acting within seconds [80]. Plasma epinephrine levels increase immediately before vasovagal reflex onset. Therefore, vasovagal-related stress just before FDG injection is considered one of the causes of increased uptake in the bilateral adrenal glands with normal adrenal configuration [81] (Fig. 17).

### Tracer extravasation

**Fig. 18:**
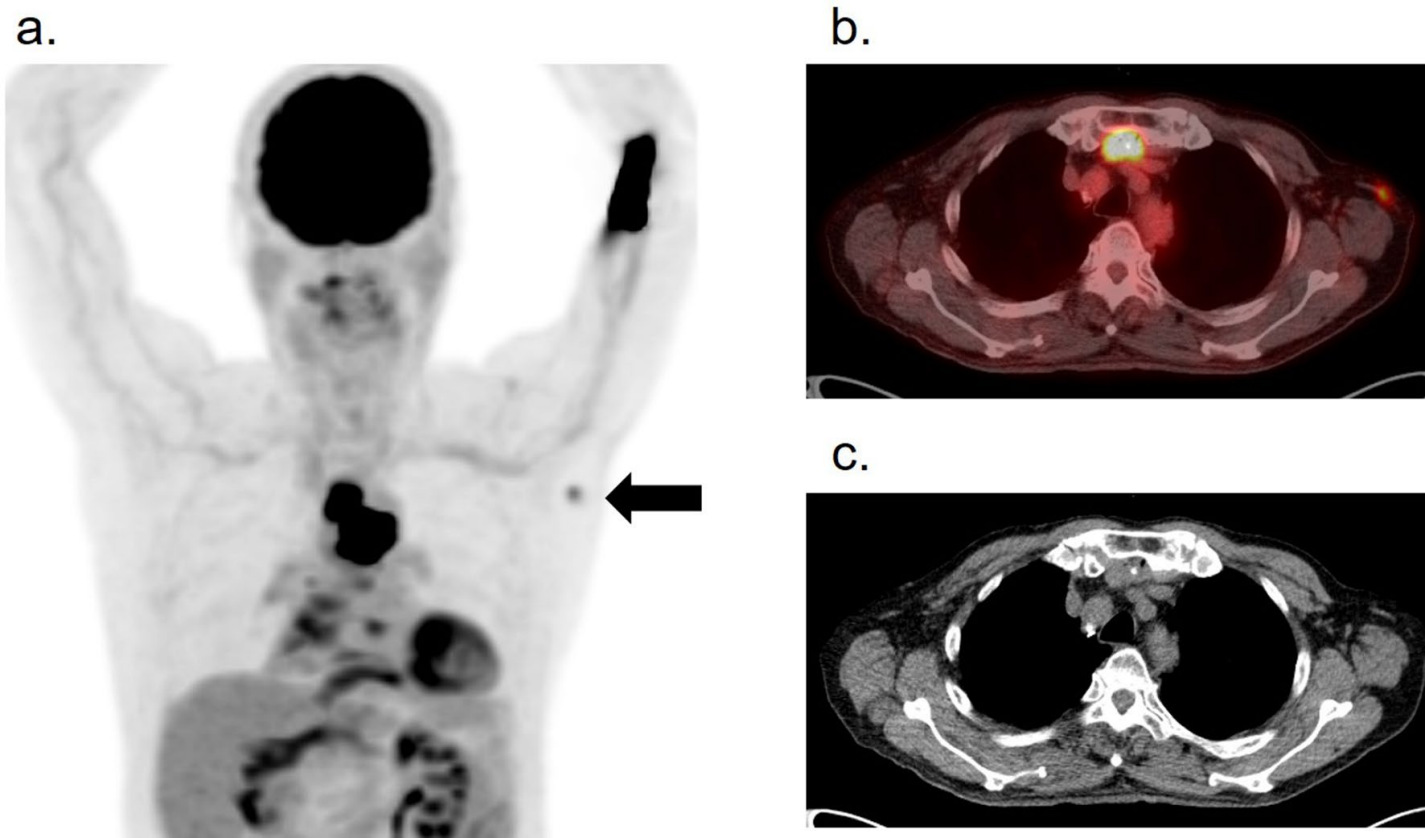
Patient with retrosternal-reconstructed esophageal cancer. A hot spot at the left axillary lymph node as a result of tracer extravasation in the ipsilateral arm is visualized. **a** MIP image, **b** axial section of the fusion image. The CT shows a normal lymph node configuration (**c**)

Radiotracer extravasation sometimes causes one or more apparent hotspots in the axillary lymph nodes on the injected side (Fig. 18). Regardless of intense FDG uptake, each node seems to be normal in size and has a fatty hilum [82]. A marked tracer distribution within the subcutaneous tissue of the upper limb around the injection site indicates tracer extravasation, even if the patients or the medical staff who administered the tracer have not been aware of it. This can cause physiological hot spots on the ipsilateral axillary area through the lymphatic system.

**Fig. 19:**
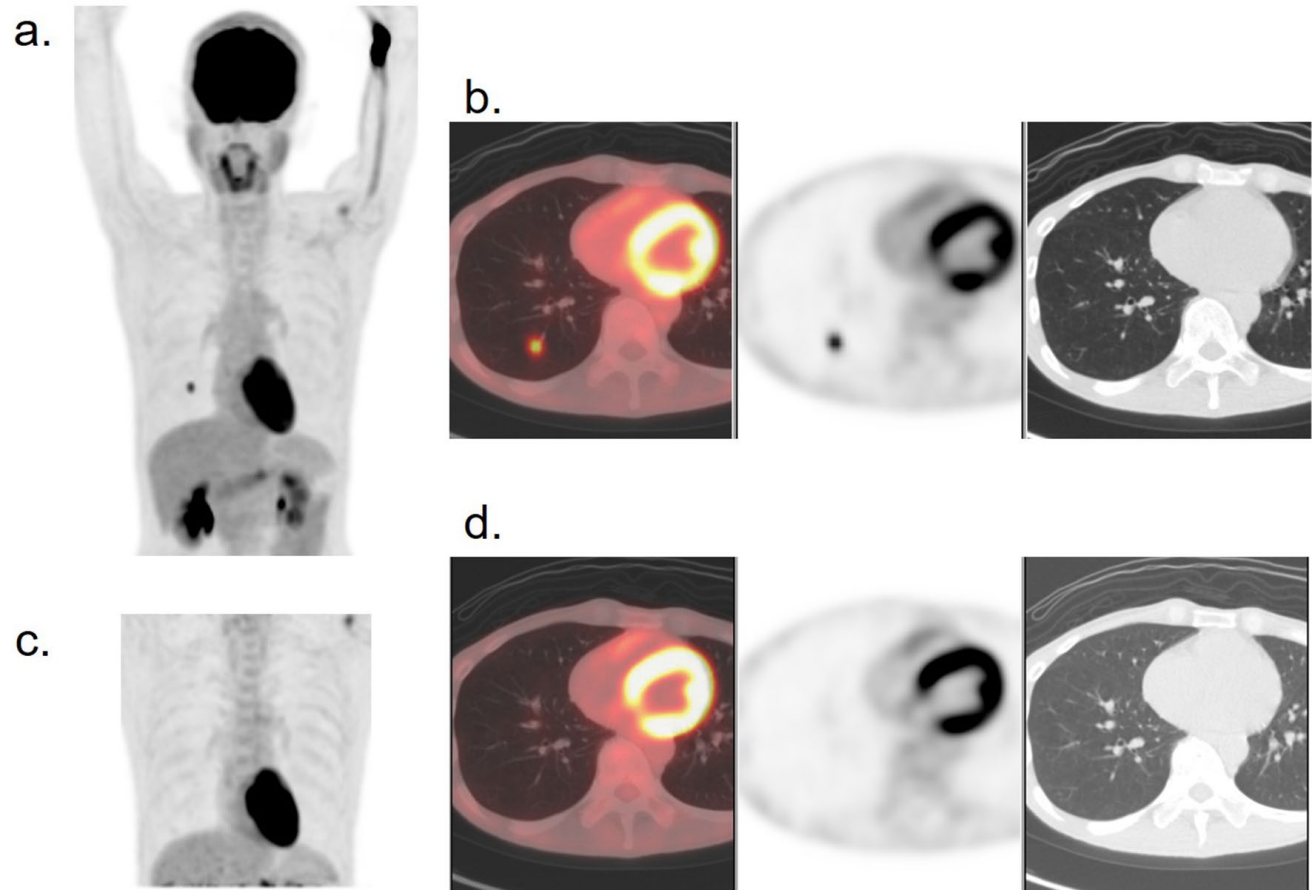
Transient hot clot in the right lung, which was considered to be a microembolism due to tracer extravasation, without abnormality on CT in the corresponding site **a** MIP, **b** axial images of fusion image (left), PET (middle) and CT (right)). The hot clot rapidly disappeared on the delayed scan that was performed 30 min later **c** MIP image, **d** axial image)

One or more transient high clot artifacts in the lung parenchyma, which are considered lung microembolisms, rarely occur when there is a tracer leak or retention in the dilated vein (Fig. 19). It is characterized by a relatively intense hot spot, without any abnormal morphological findings on CT. Delayed images usually show that the hotspot disappears or moves within a short time interval [83].

## Conclusions

FDG, which reflects glucose uptake, positively depicts many inflammatory and physiological activities, as well as tumors. Therefore, when interpreting FDG–PET results, it is necessary to consider various situations and influences. Recognizing the information on prior medical procedures and the treatment that the patient has received is important. It is also meaningful to consider whether there are any adverse effects of the medicines he/she is taking for cancer or other diseases. An unexpected finding may be related to the previous treatment or medical procedure that the attending physician had not previously recognized. In diagnosis of the PET/CT images, collecting information directly from the patient, in addition to a careful check of the medical records, should be performed diligently to avoid misdiagnosis.
